# Optical Textures and Orientational Structures in Cholesteric Droplets with Conical Boundary Conditions

**DOI:** 10.3390/molecules25071740

**Published:** 2020-04-10

**Authors:** Anna P. Gardymova, Mikhail N. Krakhalev, Victor Ya. Zyryanov

**Affiliations:** 1 Institute of Engineering Physics and Radio Electronics, Siberian Federal University, 660041 Krasnoyarsk, Russia; kmn@iph.krasn.ru; 2 Kirensky Institute of Physics, Federal Research Center KSC SB RAS, 660036 Krasnoyarsk, Russia; zyr@iph.krasn.ru

**Keywords:** cholesteric liquid crystal, droplet, optical texture, orientational structure, conical surface anchoring, topological defect

## Abstract

Cholesteric droplets dispersed in polymer with conical boundary conditions have been studied. The director configurations are identified by the polarising microscopy technique. The axisymmetric twisted axial-bipolar configuration with the surface circular defect at the droplet’s equator is formed at the relative chirality parameter $N_{0}\leq 2.9$. The intermediate director configuration with the deformed circular defect is realised at $2.9<N_{0}<3.95$, and the layer-like structure with the twisted surface defect loop is observed at $N_{0}\geq 3.95$. The cholesteric layers in the layer-like structure are slightly distorted although the cholesteric helix is untwisted.

## 1. Introduction

Recently, the droplet dispersions of liquid crystals (LCs) in a solid or liquid matrix attract more interest of researchers [1,2,3,4,5,6,7,8,9]. The optical properties of such materials depend on the orientational structures (director configurations) formed in LC droplets [6]. The configuration of director **n** (a unit vector oriented along the preferred orientation of the long axes of molecules), in turn, depends on the LC elastic constants, boundary conditions (preferred director orientation on the interface, LC anchoring energy), droplet’s size and shape, applied electric (magnetic) field [1,10,11,12]. The orientational structure and, consequently, the optical properties of such liquid crystal materials can be changed by modifying the boundary conditions [13,14,15,16,17], varying LC parameters or applying an electric field [1]. It makes it possible to use them in the electrically controlled shutters [18,19], sensors [20,21], lasers [22,23], polarizers [24,25], etc.

Cholesteric liquid crystals (CLCs) in a free state are characterised by a helical structure of the director field with an intrinsic helix pitch $p_{0}$ (the distance at which the director turns by 2π angle). The orientational structure of cholesteric in the droplets depends on the ratio of droplet diameter *d* to $p_{0}$ [26,27,28,29,30]. The relative chirality parameter $N_{0}=2d/p_{0}$ indicating the number of π turns on the droplet diameter is usually applied to analyse this dependence. For instance, at the tangential boundary conditions (the director **n** is oriented parallel to the interface) the twisted bipolar structure is formed at $N_{0}<2$ [26,27,28,31,32] and the structure with diametrical or radial dislocations is realised at $N_{0}>5$ [8,31,32,33]. A number of possible metastable configurations were theoretically examined in Ref. [29]. The influence of the electric field on the CLC orientational structures in the droplets with the tangential boundary conditions was studied in detail in Ref. [34,35].

In the cholesteric droplets with homeotropic boundary conditions (the director **n** is oriented perpendicular to the interface), the helical ordering of the director is frustrated, which leads to the formation of various orientational structures [33,35,36,37,38,39,40]. Thus, structures with point defect (hedgehog) in the bulk or near the droplet surface are observed at small $N_{0}<2.5$ [36,37]. The layer-like structure with the double twisted defect loop [33,35,38,39,40] or structures with several points defects [36,37] are formed at large $N_{0}>2.5$. When $2.9<N_{0}<5.8$, the axisymmetric structure with the surface circular defect at the droplet’s equator is formed [41,42].

Currently, the cholesteric droplets with conical boundary conditions (director **n** is tilted to the interface by the angle $0^{{}^{\circ}}<\theta_{0}<90^{{}^{\circ}}$) have not been sufficiently studied. It is known that a weak conical anchoring appears at the interface of LC and own isotropic phase. In this case, the axisymmetric ($C_{\infty }$) double twisted structure ($N_{0}<2$) or the defect-free structure with the uniform helix axis distribution ($N_{0}>2$) have been observed in cholesteric droplets [43,44,45,46]. Recently, we have obtained and investigated the nematic droplets dispersed in polymer with the conical boundary conditions [47]. Several orientational structures differing by the type and relative arrangement of surface and bulk topological defects are formed in such droplets [12].

In the present paper the orientational structures of the cholesteric droplets dispersed in polymer with conical boundary conditions have been studied.

## 2. Results and Discussion

### 2.1. Twisted Axial-Bipolar Structure

**Figure 1:**
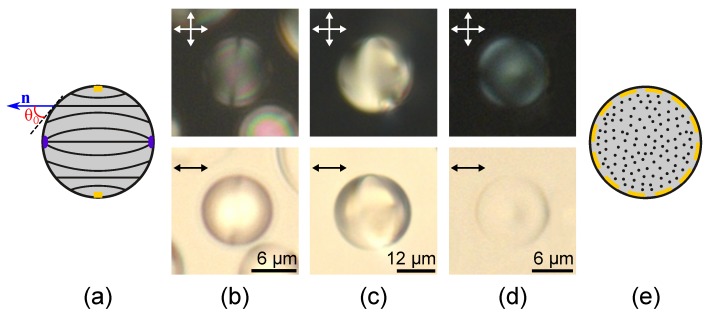
The nematic LN-396 droplets with the axial-bipolar configuration. Scheme of the director field in the droplet central section passing through the bipolar axis (**a**). POM (polarising optical microscopy) photos of the droplets with the bipolar axis oriented parallel (**b**), at approximately 60${}^{{}^{\circ}}$ angle (**c**) and perpendicular (**d**) to the sample plane taken in the crossed polarisers (top row) and without analyser (bottom row). Scheme of the director field in the central section perpendicular to the bipolar axis (**e**). Violet semicircles indicate the surface point defects, the orange rectangles indicate the sections of circular defect in (**a**), and the orange dashed line indicates the circular defect in (**e**). Hereinafter, the orientation of polarisers is indicated by the double arrows.

At the conical boundary conditions the axial-bipolar configuration is the most frequently realised within the droplets of LN-396 nematic [12]. This structure is characterised by two surface point defects (boojums) located at the diametrically opposite poles of the droplet and the surface circular defect at the droplet’s equator (Figure 1). The specific feature of the axial-bipolar configuration is a random orientation of the bipolar axis relative to the short axis of oblate droplets (the normal to the composite film plane) formed in the sample. For this reason, the various optical textures of nematic droplets with the axial-bipolar structure are observed [47,48].

**Figure 2:**
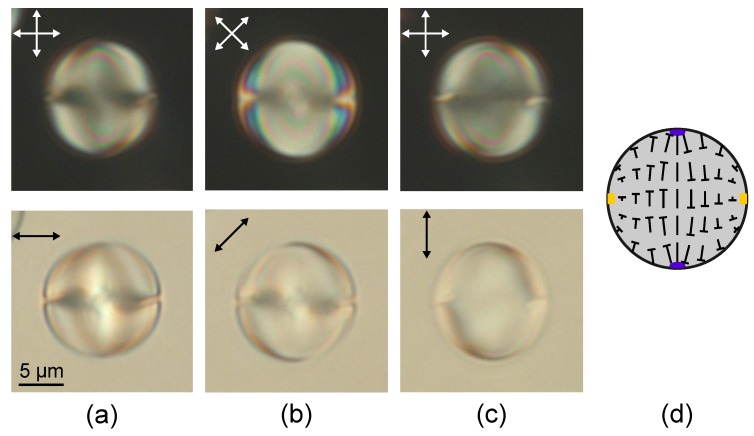
POM photos of the cholesteric droplets at $N_{0}=2.9$ taken in the crossed polarisers (top row) and without analyser (bottom row). The circular defect plane is perpendicular to the film plane. The polariser is oriented parallel (**a**), at angle 45${}^{{}^{\circ}}$ (**b**) and perpendicular (**c**) to the circular defect plane. Scheme of the director orientation in the central droplet section (**d**).

**Figure 3:**
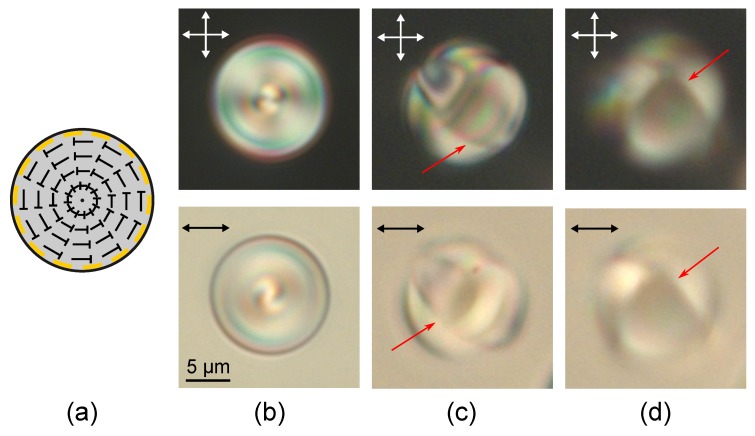
The cholesteric droplets at $N_{0}=2.9$. Scheme of the director orientation in the central cross section of droplet with the plane of circular defect parallel to film plane (**a**). POM photos of CLC droplets with the circular defect plane oriented parallel (**b**), at approximately 50${}^{{}^{\circ}}$ angle (**c**), (**d**) to the film plane taken in the crossed polarisers (top row) and without analyser (bottom row). The microscope is focused on the upper (**c**) and lower (**d**) part of the circular defect. Single arrows indicate a position of the linear defect.

The axisymmetric twisted axial-bipolar configuration is formed in the cholesteric droplets at $N_{0}\leq 2.9$ under conical surface anchoring (Figure 2 and Figure 3). The director twist angle on the droplet diameter in the equator plane (the plane of the circular defect) depends on $N_{0}$ and can be measured by the method of rotating polariser and analyser [49]. For instance, the twist angle is 130${}^{{}^{\circ}}\pm 5^{{}^{\circ}}$ for droplets with $N_{0}=2.2$, 160${}^{{}^{\circ}}\pm 5^{{}^{\circ}}$ at $N_{0}=2.5$, and 180${}^{{}^{\circ}}\pm 5^{{}^{\circ}}$ at $N_{0}=2.9$. Like in nematic droplets, the symmetry axis of the twisted axial-bipolar droplets is oriented differently relative to the sample plane (Figure 2 and Figure 3). Figure 3b shows the droplet in which the circular defect is situated in the film plane and Figure 3c,d demonstrate the droplet with the symmetry axis tilted to the film plane by an angle approximately 50${}^{{}^{\circ}}$. In the last case, the circular surface defect and two boojums located above and below the central cross-section of droplet can be clearly observed by changing a position of the microscope focus [47].

### 2.2. Layer-Like Structure

**Figure 4:**
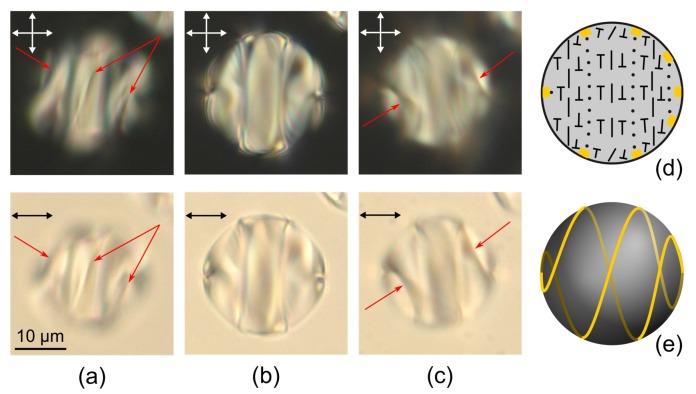
POM photos of cholesteric droplets at $N_{0}=5.2$ taken in the crossed polarisers (top row) and without analyser (bottom row). The microscope is focused on the linear defect above the droplet centre (**a**), on the droplet centre (**b**) and on the linear defect below the droplet centre (**c**). Schemes of the director orientation in the central section of the droplet (**d**) and the twisted defect loop on the droplet surface (**e**). Single arrows indicate a position of the linear defect.

The layer-like structure is formed in the cholesteric droplets under both tangential and homeotropic boundary conditions at sufficiently high $N_{0}$ [38,39]. In CLC droplets under conical boundary conditions the layer-like structure is observed at $N_{0}\geq 3.95$ (Figure 4). The sharp isoclinic lines are revealed when the cholesteric layers are orthogonal to the film plane in the central cross-section of CLC droplet (Figure 4). These lines correspond to the areas in the cross-section of droplet where the director is oriented parallel to the microscope axis [38]. The layer-like structure is characterised by minor deformation of cholesteric layers (isoclinic lines are slightly curved), and the number *N* of π director turns on the droplet diameter is less than $N_{0}$ (the effect of cholesteric helix untwisting [50]). In this case, the discrepancy between *N* and $N_{0}$ decreases as the droplet’s diameter increases. The values of *N* and $N_{0}$ for different sizes of CLC droplets are presented in Table 1. The droplets with characteristic optical textures at *N* close to the integer value were chosen for measurement [50]. It is seen that $N_{0}$ is approximately twice the value of *N* for the droplet of d=19.2 μm, and the ratio of $N_{0}$ to the corresponding N=6 (d=43.3 μm) is 1.49. Thus, as the droplet’s diameter increases the ratio $N_{0}/N$ decreases more slowly than in the droplets under the strong homeotropic anchoring [50]. Apparently, this effect is due to quite weak polar anchoring strength of LC with polymer $W_{0}∼10^{-6}$ J/m${}^{2}$ [47]. This polar surface anchoring corresponds to a surface extrapolation length $d_{e}=K/W_{0}$ (*K* is the Frank elastic constant) of the order of several microns [10,51]. Accordingly, the balance between the surface and elastic Frank energy should be very sensitive to the elastic constants value, the intrinsic helix pitch $p_{0}$, and the droplets’ size in the range from several microns to tens of microns. As a result, the dependence of the forming structure on the droplet size (in our case, the number *N* of the layer-like structure) should be more complicated compared to the case of strong anchoring.

**Figure 5:**
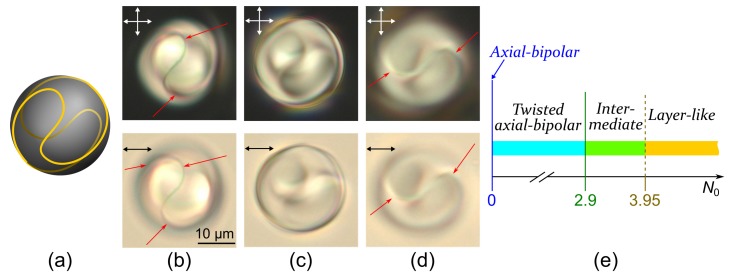
Scheme of the twisted defect loop on the droplet surface (**a**). POM photos of cholesteric droplets at $N_{0}=5.2$ taken in the crossed polarisers (top row) and without analyser (bottom row). The microscope is focused on the linear defect above the droplet centre (**b**), on the droplet centre (**c**) and on the linear defect below the droplet centre (**d**). (**e**) Diagram of the droplet state observed experimentally at various droplet sizes expressed by the relative helicity parameter $N_{0}$. Single arrows indicate the position of the linear defect.

A formation of the layer-like structure leads to either a local disturbance of the boundary conditions, as in the case of weak surface anchoring [45] or an appearance of the linear surface defect as in the case of strong homeotropic anchoring [38]. The double twisted defect loop is formed near the droplets surface under study (Figure 4 and Figure 5). The linear surface defect transverse the central droplet section at the points located at the isoclinic lines, therefore the director is parallel to the interface near the linear defect. If the cholesteric axis is oriented mainly perpendicular to the film plane, the cholesteric layers are practically invisible (Figure 5c). When changing the microscope focus, the double twisted defect loop can be seen (Figure 5).

**Figure 6:**
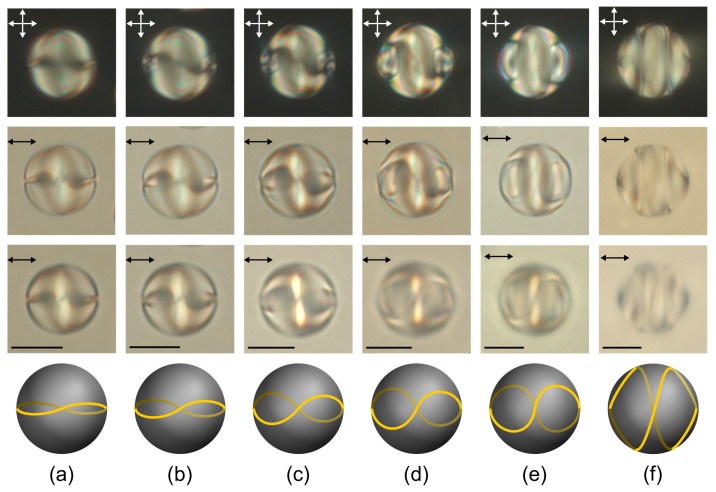
POM photos of cholesteric droplets at $N_{0}=3.05$ (**a**), $N_{0}=3.25$ (**b**), $N_{0}=3.60$ (**c**), $N_{0}=3.71$ (**d**), $N_{0}=3.92$ (**e**), and $N_{0}=3.98$ (**f**) taken in the crossed polarisers (first row) and without analyser when the microscope is focused on the centre (second row) and above the droplet centre (third row). Schemes of the twisted defect loops on the droplet surface (fourth row). Scale bars are 10 μm.

The twisted axial-bipolar structure ($N_{0}<2.9$) has the $C_{\infty }$ symmetry axis, and the layer-like configuration ($N_{0}>3.95$) has the $C_{2}$ symmetry axis. A transition from one configuration to another one proceeds smoothly as $N_{0}$ increases (Figure 5e). The intermediate structures with the defect line deformed around the droplet’s equator are observed at $2.9<N_{0}<3.95$. At that, the deformation degree of this line increases as $N_{0}$ rises up to 3.95 (Figure 6). The observed structures and corresponding $N_{0}$ ranges are stable. In this case, the position of the intervals’ boundaries (Figure 5e) should be dependent on the anchoring energy value and/or the intrinsic helix pitch $p_{0}$. For instance, in the work of Yoshioka et al. [45], the transition between an axisymmetric double twisted structure ($C_{\infty }$) and a layer-like structure occurs at $N_{0}≅2.1$ without the formation of intermediate structures.

## 3. Materials and Methods

The nematic mixture LN-396 (Belarusian State Technological University, Minsk, Belarus) doped with the left-handed chiral additive cholesteryl acetate (Sigma Aldrich, St. Louis, MO, USA) was used as a cholesteric. The concentration of cholesteryl acetate was 1.5%, that corresponds to $p_{0}$ = 9.7 μm [52]. The cholesteric was dispersed in thermoplastic poly(isobutyl methacrylate) (PiBMA) (Sigma Aldrich, St. Louis, MO, USA) having the glass transition temperature $T_{g}=65{\;}^{{}^{\circ}}$C. The samples were made by solvent-induced phase separation technique [1]. CLC was added to the 4% solution of polymer and butyl acetate. The weight ratio of the components was CLC:PiBMA = 50:50. The prepared homogeneous solution was poured on the glass substrate and dried in the Petri dish for 24 h. In the result, the polymer dispersed cholesteric liquid crystal films were formed. The average film thickness was 40 μm and the droplet size *d* was varied in the range of 7–45 μm. PiBMA specifies for the nematic mixture LN-396 the conical boundary conditions with the director tilt angle at the interface $\theta_{0}=50^{{}^{\circ}}$ [47]. The CLC droplets were studied by means of the polarising optical microscope (POM) Axio Imager.A1m (Carl Zeiss AG, Oberkochen, Germany) at the temperature $t=25{\;}^{{}^{\circ}}$C.

## 4. Conclusions

The orientational structures in cholesteric droplets with the unique conical boundary conditions with the director tilt angle $\theta_{0}=50^{{}^{\circ}}$ have been studied. In such droplets, various combinations of topological features were revealed that are inherent to both nematic droplets with conical anchoring [47] and cholesteric droplets with homeotropic boundary conditions [42]. The axisymmetric twisted axial-bipolar configuration characterised by two boojums and the surface circular defect at the droplet’s equator is formed at $N_{0}\leq 2.9$. It has been shown that the orientational structure intermediate between axisymmetric and layer-like is realised at $2.9<N_{0}<3.95$. In such structure, the defect ring is deformed around the droplet’s equator. The layer-like structure with a minor deformation of cholesteric layers and the twisted defect loop on droplet’s surface is formed at $N_{0}\geq 3.95$. The untwisting of the cholesteric helix is observed in the layer-like structure, at that the discrepancy between *N* and $N_{0}$ decreases as the droplet diameter increases more slowly than in the droplets with the strong homeotropic anchoring [50]. Apparently, the effect is due to the quite weak polar energy of conical anchoring $W_{0}∼10^{-6}$ J/m${}^{2}$. As a result, both the value of $N_{0}$ and the size of the droplet should affect the formed structure similar to the case of CLC in the flat layer with tangential-conical boundary conditions, where the orientational structure depends not only on the ratio of layer thickness to the intrinsic helix pitch but also the CLC layer thickness [52]. The composite material under study can be interesting for the development of CLC dispersed systems in which the layer structure uniformity is an important factor, for instance, for optical lasing [53].

## Figures and Tables

**Table 1:** The values of *N* and $N_{0}$ for the different droplet diameters *d*.

d, μ **m**	19.2	24.7	30.1	36.8	43.3
N	2.0	3.0	4.0	5.0	6.0
$N_{0}$	3.96	5.09	6.21	7.59	8.93

## References

[1] Drzaic P.S. (1995). Liquid Crystal Dispersions.

[2] Urbanski M., Reyes C.G., Noh J., Sharma A., Geng Y., Subba Rao Jampani V., Lagerwall J.P.F. (2017). Liquid crystals in micron-scale droplets, shells and fibers. J. Phys. Condens. Matter.

[3] Lancia F., Yamamoto T., Ryabchun A., Yamaguchi T., Sano M., Katsonis N. (2019). Reorientation behavior in the helical motility of light-responsive spiral droplets. Nat. Commun..

[4] Shvetsov S., Orlova T., Emelyanenko A.V., Zolot’ko A. (2019). Thermo-optical generation of particle-like structures in frustrated chiral nematic film. Crystals.

[5] Lopez-Leon T., Fernandez-Nieves A. (2011). Drops and shells of liquid crystal. Colloid Polym. Sci..

[6] Kitzerow H.S. (1994). Polymer-dispersed liquid crystals from the nematic curvilinear aligned phase to ferroelectric films. Liq. Cryst..

[7] Darmon A., Benzaquen M., Čopar S., Dauchot O., Lopez-Leon T. (2016). Topological defects in cholesteric liquid crystal shells. Soft Matter.

[8] Tran L., Lavrentovich M.O., Beller D.A., Li N., Stebe K.J., Kamien R.D. (2016). Lassoing saddle splay and the geometrical control of topological defects. Proc. Natl. Acad. Sci. USA.

[9] Tran L., Kim H.N., Li N., Yang S., Stebe K.J., Kamien R.D., Haase M.F. (2018). Shaping nanoparticle fingerprints at the interface of cholesteric droplets. Sci. Adv..

[10] Erdmann J.H., Žumer S., Doane J.W. (1990). Configuration transition in a nematic liquid crystal confined to a small spherical cavity. Phys. Rev. Lett..

[11] Rudyak V.Y., Emelyanenko A.V., Loiko V.A. (2013). Structure transitions in oblate nematic droplets. Phys. Rev. E.

[12] Rudyak V.Y., Krakhalev M.N., Prishchepa O.O., Sutormin V.S., Emelyanenko A.V., Zyryanov V.Y. (2017). Orientational structures in nematic droplets with conical boundary conditions. JETP Lett..

[13] Prishchepa O.O., Shabanov A.V., Zyryanov V.Y. (2005). Director configurations in nematic droplets with inhomogeneous boundary conditions. Phys. Rev. E.

[14] Zyryanov V.Y., Krakhalev M.N., Prishchepa O.O., Shabanov A.V. (2007). Orientational structure transformations caused by the electric-field-induced ionic modification of the interface in nematic droplets. JETP Lett..

[15] Zyryanov V.Y., Krakhalev M.N., Prishchepa O.O., Shabanov A.V. (2008). Inverse regime of ionic modification of surface anchoring in nematic droplets. JETP Lett..

[16] Gupta J.K., Abbott N.L. (2009). Principles for manipulation of the lateral organization of aqueous-soluble surface-active molecules at the liquid crystal-aqueous interface. Langmuir.

[17] Krakhalev M.N., Sutormin V.S., Prishchepa O.O., Kuz’menok N.M., Mikhalyonok S.G., Bezborodov V.S., Zyryanov V.Y. (2019). Anionic-cationic surfactant mixture providing the electrically controlled homeotropic surface anchoring of liquid crystals. J. Mol. Liq..

[18] Doane J.W., Vaz N.A., Wu B., Žumer S. (1986). Field controlled light scattering from nematic microdroplets. Appl. Phys. Lett..

[19] Bouteiller L., Lebarny P. (1996). Polymer-dispersed liquid crystals: Preparation, operation and application. Liq. Cryst..

[20] Manna U., Zayas-Gonzalez Y.M., Carlton R.J., Caruso F., Abbott N.L., Lynn D.M. (2013). Liquid crystal chemical sensors that cells can wear. Angew. Chem. Int. Ed..

[21] Wang Y., Zhao L., Xu A., Wang L., Zhang L., Liu S., Liu Y., Li H. (2018). Detecting enzymatic reactions in penicillinase via liquid crystal microdroplet-based pH sensor. Sens. Actuators B Chem..

[22] Wang Y., Li H., Zhao L., Liu Y., Liu S., Yang J. (2016). Tunable whispering gallery modes lasing in dye-doped cholesteric liquid crystal microdroplets. Appl. Phys. Lett..

[23] Humar M. (2016). Liquid-crystal-droplet optical microcavities. Liq. Cryst..

[24] Bloisi F., Ruocchio C., Terrecuso P., Vicari L. (1996). Optoelectronic polarizer by PDLC. Liq. Cryst..

[25] Krakhalev M.N., Prishchepa O.O., Sutormin V.S., Zyryanov V.Y. (2019). Polymer dispersed nematic liquid crystal films with conical boundary conditions for electrically controllable polarizers. Opt. Mater..

[26] Xu F., Crooker P.P. (1997). Chiral nematic droplets with parallel surface anchoring. Phys. Rev. E.

[27] Zhou Y., Bukusoglu E., Martínez-González J.A., Rahimi M., Roberts T.F., Zhang R., Wang X., Abbott N.L., de Pablo J.J. (2016). Structural transitions in cholesteric liquid crystal droplets. ACS Nano.

[28] Gardymova A.P. (2015). Orientation structures of the chiral nematic droplets in a polymer matrix. Liq. Cryst. Their Appl..

[29] Seč D., Porenta T., Ravnik M., Žumer S. (2012). Geometrical frustration of chiral ordering in cholesteric droplets. Soft Matter.

[30] Prishchepa O.O., Zyryanov V.Y., Gardymova A.P., Shabanov V.F. (2008). Optical textures and orientational structures of nematic and cholesteric droplets with heterogeneous boundary conditions. Mol. Cryst. Liq. Cryst..

[31] Bezić J., Žumer S. (1992). Structures of the cholesteric liquid crystal droplets with parallel surface anchoring. Liq. Cryst..

[32] Kurik M.V., Lavrentovich O.D. (1982). Negative-positive monopole transitions in cholesteric liquid crystals. JETP Lett..

[33] Bouligand Y., Livolant F. (1984). The organization of cholesteric spherulites. J. Phys..

[34] Yang D.K., Crooker P.P. (1991). Field-induced textures of polymer-dispersed chiral liquid crystal microdroplets. Liq. Cryst..

[35] Kitzerow H.S., Crooker P. (1993). Electric field effects on the droplet structure in polymer dispersed cholesteric liquid crystals. Liq. Cryst..

[36] Posnjak G., Čopar S., Muševič I. (2016). Points, skyrmions and torons in chiral nematic droplets. Sci. Rep..

[37] Posnjak G., Čopar S., Muševič I. (2017). Hidden topological constellations and polyvalent charges in chiral nematic droplets. Nat. Commun..

[38] Krakhalev M.N., Gardymova A.P., Prishchepa O.O., Rudyak V.Y., Emelyanenko A.V., Liu J.H., Zyryanov V.Y. (2017). Bipolar configuration with twisted loop defect in chiral nematic droplets under homeotropic surface anchoring. Sci. Rep..

[39] Seč D., Čopar S., Žumer S. (2014). Topological zoo of free-standing knots in confined chiral nematic fluids. Nat. Commun..

[40] Orlova T., Asshoff S.J., Yamaguchi T., Katsonis N., Brasselet E. (2015). Creation and manipulation of topological states in chiral nematic microspheres. Nat. Commun..

[41] Krakhalev M.N., Rudyak V.Y., Gardymova A.P., Zyryanov V.Y. (2019). Toroidal configuration of a cholesteric liquid crystal in droplets with homeotropic anchoring. JETP Lett..

[42] Krakhalev M.N., Rudyak V.Y., Prishchepa O.O., Gardymova A.P., Emelyanenko A.V., Liu J.H., Zyryanov V.Y. (2019). Orientational structures in cholesteric droplets with homeotropic surface anchoring. Soft Matter.

[43] Oswald P. (2009). Lehmann rotation of cholesteric droplets subjected to a temperature gradient: Role of the concentration of chiral molecules. Eur. Phys. J. E.

[44] Ito F., Yoshioka J., Tabe Y. (2016). Heat-driven rotation in cholesteric droplets with a double twisted structure. J. Phys. Soc. Jpn..

[45] Yoshioka J., Ito F., Tabe Y. (2016). Stability of a double twisted structure in spherical cholesteric droplets. Soft Matter.

[46] Poy G., Bunel F., Oswald P. (2017). Role of anchoring energy on the texture of cholesteric droplets: Finite-element simulations and experiments. Phys. Rev. E.

[47] Krakhalev M.N., Prishchepa O.O., Sutormin V.S., Zyryanov V.Y. (2017). Director configurations in nematic droplets with tilted surface anchoring. Liq. Cryst..

[48] Rudyak V.Y., Krakhalev M.N., Sutormin V.S., Prishchepa O.O., Zyryanov V.Y., Liu J.H., Emelyanenko A.V., Khokhlov A.R. (2017). Electrically induced structure transition in nematic liquid crystal droplets with conical boundary conditions. Phys. Rev. E.

[49] Drzaic P.S. (1999). A case of mistaken identity: Spontaneous formation of twisted bipolar droplets from achiral nematic materials. Liq. Cryst..

[50] Krakhalev M.N., Gardymova A.P., Emel’yanenko A.V., Liu J.H., Zyryanov V.Y. (2017). Untwisting of the helical structure of cholesteric droplets with homeotropic surface anchoring. JETP Lett..

[51] Rofouie P., Pasini D., Rey A.D. (2017). Morphology of elastic nematic liquid crystal membranes. Soft Matter.

[52] Krakhalev M.N., Bikbaev R.G., Sutormin V.S., Timofeev I.V., Zyryanov V.Y. (2019). Nematic and cholesteric liquid crystal structures in cells with tangential-conical boundary conditions. Crystals.

[53] Belmonte A., Ussembayev Y.Y., Bus T., Nys I., Neyts K., Schenning A.P.H.J. (2020). Dual Light and Temperature Responsive Micrometer-Sized Structural Color Actuators. Small.

